# Advances in the Total Synthesis of Aflatoxins

**DOI:** 10.3389/fchem.2021.779765

**Published:** 2021-11-30

**Authors:** Liyan Yang, Zhonglei Wang

**Affiliations:** ^1^ School of Physics and Physical Engineering, Qufu Normal University, Qufu, China; ^2^ Key Laboratory of Green Natural Products and Pharmaceutical Intermediates in Colleges and Universities of Shandong Province, School of Chemistry and Chemical Engineering, Qufu Normal University, Qufu, China; ^3^ Key Laboratory of Life-Organic Analysis of Shandong Province, School of Chemistry and Chemical Engineering, Qufu Normal University, Qufu, China; ^4^ School of Pharmaceutical Sciences, Tsinghua University, Beijing, China

**Keywords:** aflatoxins, natural products, contaminants, total syntheses, formal syntheses

## Abstract

**Abstract:** Aflatoxins, which are produced by *Aspergillus flavus*, *Aspergillus nomius*, and *Aspergillus parasiticus*, are a group of pentacyclic natural products with difuran and coumarin skeletons. They mainly include aflatoxin B_1_, B_2_, G_1_, G_2_, M_1_, and M_2_. Biologically, aflatoxins are of concern to human health as they can be present as contaminants in food products. The unique skeletons of aflatoxins and their risk to human health have led to the publication of nine remarkable total syntheses (including three asymmetric syntheses) and ten formal total syntheses (including four asymmetric formal syntheses) of aflatoxins in the past 55 years. To better understand the mechanism of the biological activity of aflatoxins and their presence in samples from the food industry, this review summarizes progress in the total synthesis of aflatoxins.

## 1 Introduction

**FIGURE 1:**
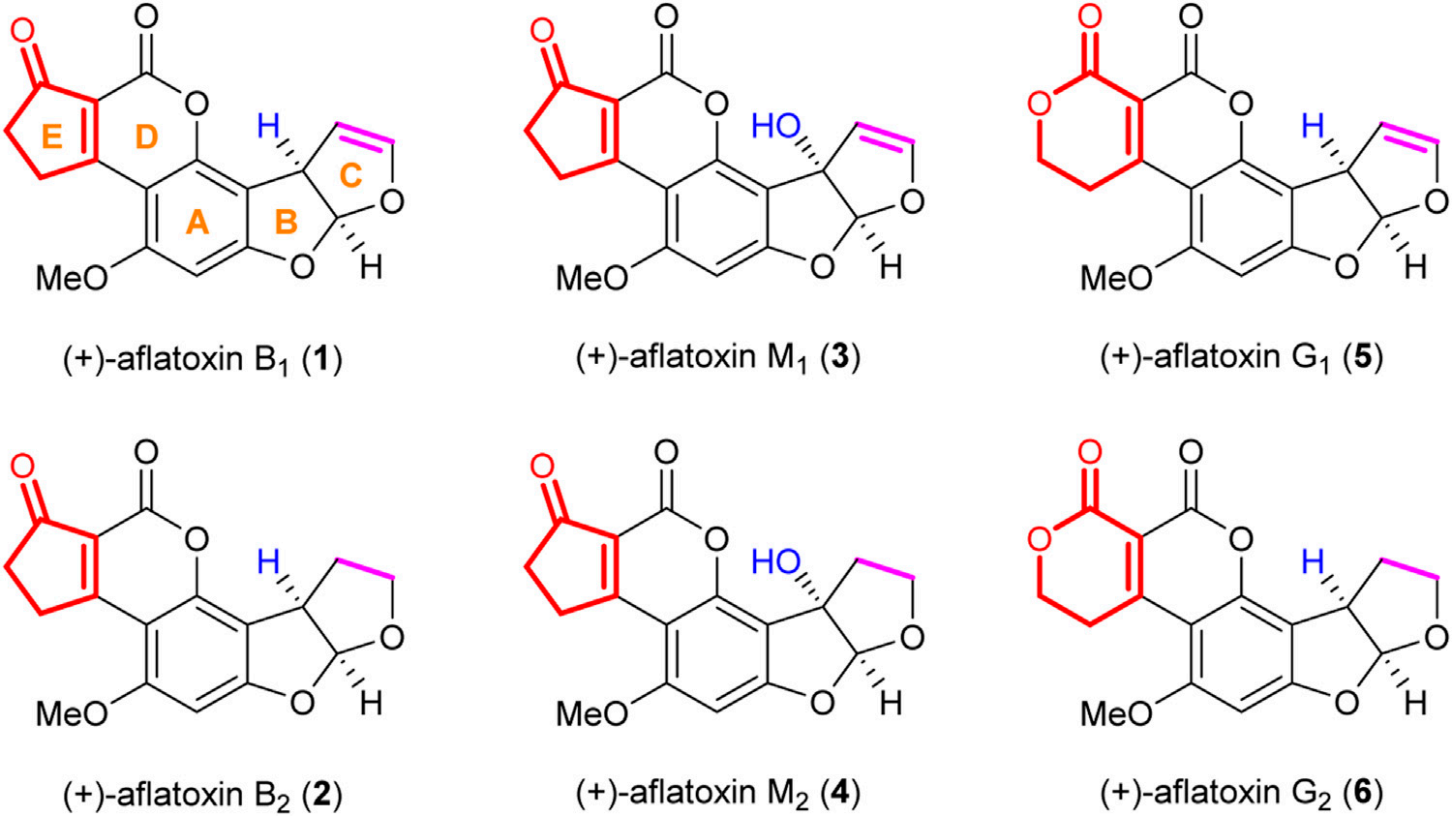
Structures of representative aflatoxins.

Aflatoxins (Figure 1) are a group of potent hepatocarcinogenic polyketide natural products produced by the fungi *Aspergillus flavus* and *Aspergillus parasiticus* (Groopman et al., 2008; Bräse et al., 2009; Wu, 2014). Aflatoxin B_1_ **1**) and G_1_ **5**) were first isolated together with aflatoxin B_2_ **2**) and G_2_ **6**) in 1963 (Hartley et al., 1963), and their structures were revealed in 1963 (G_1_ and B_1_) (Asao et al., 1963) and 1965 (G_2_ and B_2_) (Asao et al., 1965) by the group of Büchi. The absolute stereochemistry of the above four aflatoxins (G_1_, B_1_, G_2_, and B_2_) was determined by chemical degradation by Büchi’s group (Brechbühler-Bader et al., 1967). Aflatoxin M_1_ **3**) and M_2_ **4**) are hydroxylated metabolites of aflatoxin B_1_ and B_2_ (Bianco et al., 2012; Lee et al., 2017). Biologically, aflatoxins present a significant risk to human health as they can be present as contaminants in food products (Roze et al., 2013; Bashiry et al., 2021; Ismail et al., 2021; Mollayusefian et al., 2021). Aflatoxins are classified as hepatocarcinogens; however, their effects on tissues other than the liver are mainly unclear (Wang et al., 2015; Schrenk et al., 2020). Thus, there is a need to obtain analytical samples from the food industry and understand the mechanism underlying the biological activity of aflatoxins.

Given the broad public health implications of aflatoxins, considerable progress has been made in the chemical synthesis of aflatoxins since the first synthesis of racemic aflatoxins by the groups of Büchi and Roberts in the 1960s (Büchi et al., 1966). The mechanisms of the biological activities of many complex natural products, including aflatoxins, remain unknown due to the impracticality of isolating the products from their natural sources; the only alternative to obtaining the natural products is practical total synthesis. Thus, to better understand the effects of aflatoxins, this article reviews important developments in the organic total synthesis of aflatoxins during the past 55 years.

## 2 Total Synthesis of Racemic Aflatoxins

### 2.1 First Total Synthesis of (±)-Aflatoxin B_1_ by Büchi Group

The Büchi group has made outstanding contributions to the chemical total synthesis of aflatoxins. This group has completed several total syntheses of challenging molecules in the aflatoxin family. These syntheses are characterized by Pechmann condensation and cascade reduction rearrangement.

**FIGURE 2:**
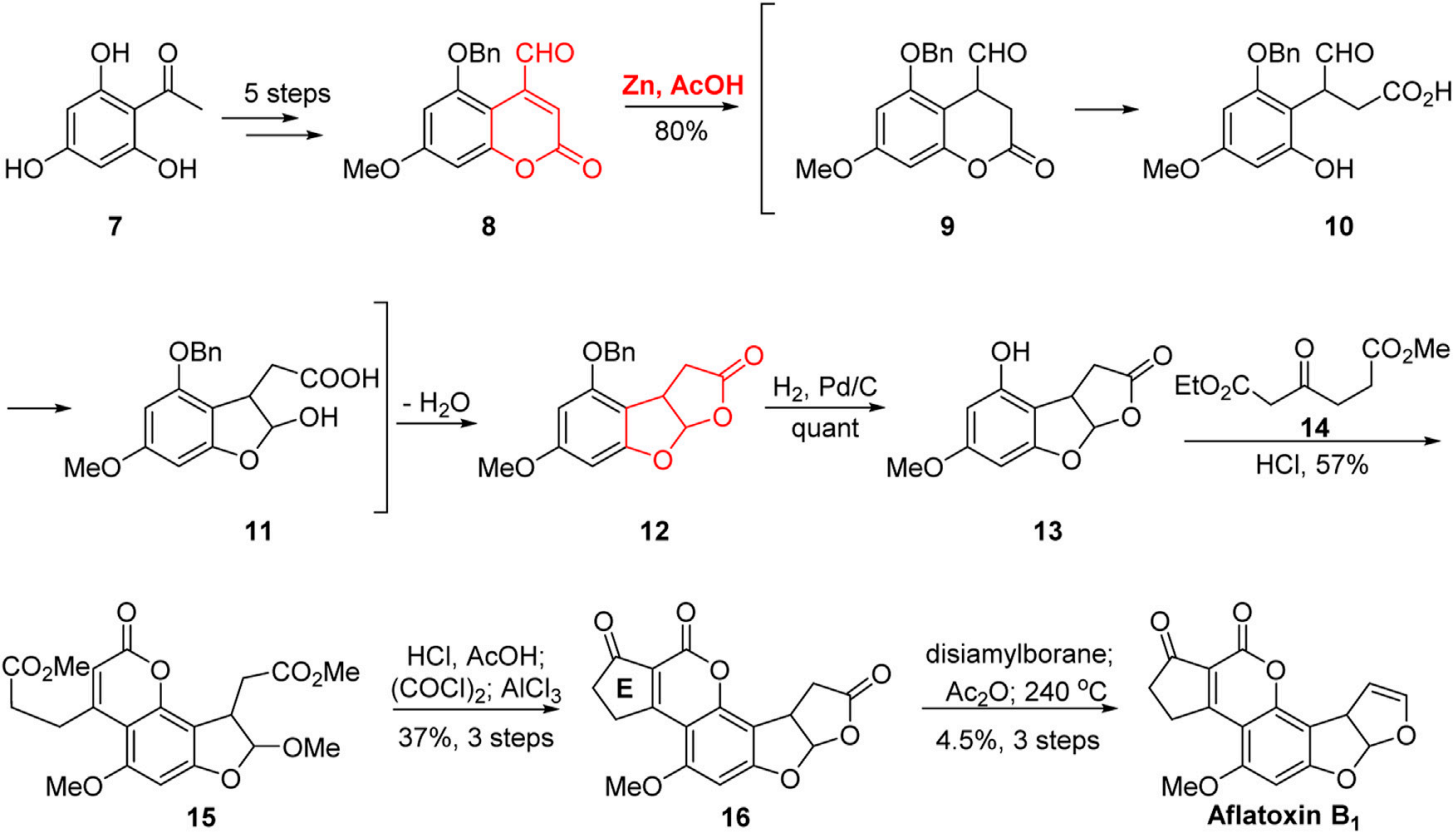
First total synthesis of (±)-aflatoxin B_1_ by Büchi group.

As early as 1966, Büchi’s group (Büchi et al., 1966; Büchi et al., 1967) completed the first total synthesis of aflatoxin B_1_, as shown in Figure 2. The aldehyde **8** was obtained from acetyl benzene **7** through five steps: non-selective acylation, methylation, deacylation, selective benzylation, veticilienylation, and allyl oxidation. In the presence of Zn/AcOH, the tricyclic skeleton **12** was efficiently constructed. The cascade reaction proceeded in three steps: 1) the reduction of the double bond of coumarin **8** in the presence of Zn/AcOH; 2) the ring opening of the lactone under the action of glacial acetic acid; and 3) the formation of a hemiacetal between the free phenol and aldehyde groups. The construction of the tricyclic framework was then completed via esterification reaction followed by the removal of the benzyl protecting group to realize the tricyclic intermediate **13**.

Next, in the presence of hydrochloric acid in methanol, the D-ring was constructed via Pechmann condensation reaction with the *β*-keto ester **14**. Notably, the C-ring was opened in the presence of hydrochloric acid in methanol. Subsequently, under the action of hydrochloric acid and acetic acid, the two ester groups underwent acetal methyl hydrolysis, leading to the re-cyclization of the C-ring. After the activation of the carboxyl group, the E-ring **16** was constructed via Friedel–Crafts reaction catalyzed by AlCl_3_. Aflatoxin B_1_ was synthesized by the selective reduction of the C-ring, acylation of the hemiacetal hydroxyl, and pyrolysis at 240°C. The first total synthesis of aflatoxin B_1_ was completed in 13 steps with a 0.9% total yield.

### 2.2 Total Synthesis of (±)-Aflatoxin B_2_

**FIGURE 3:**
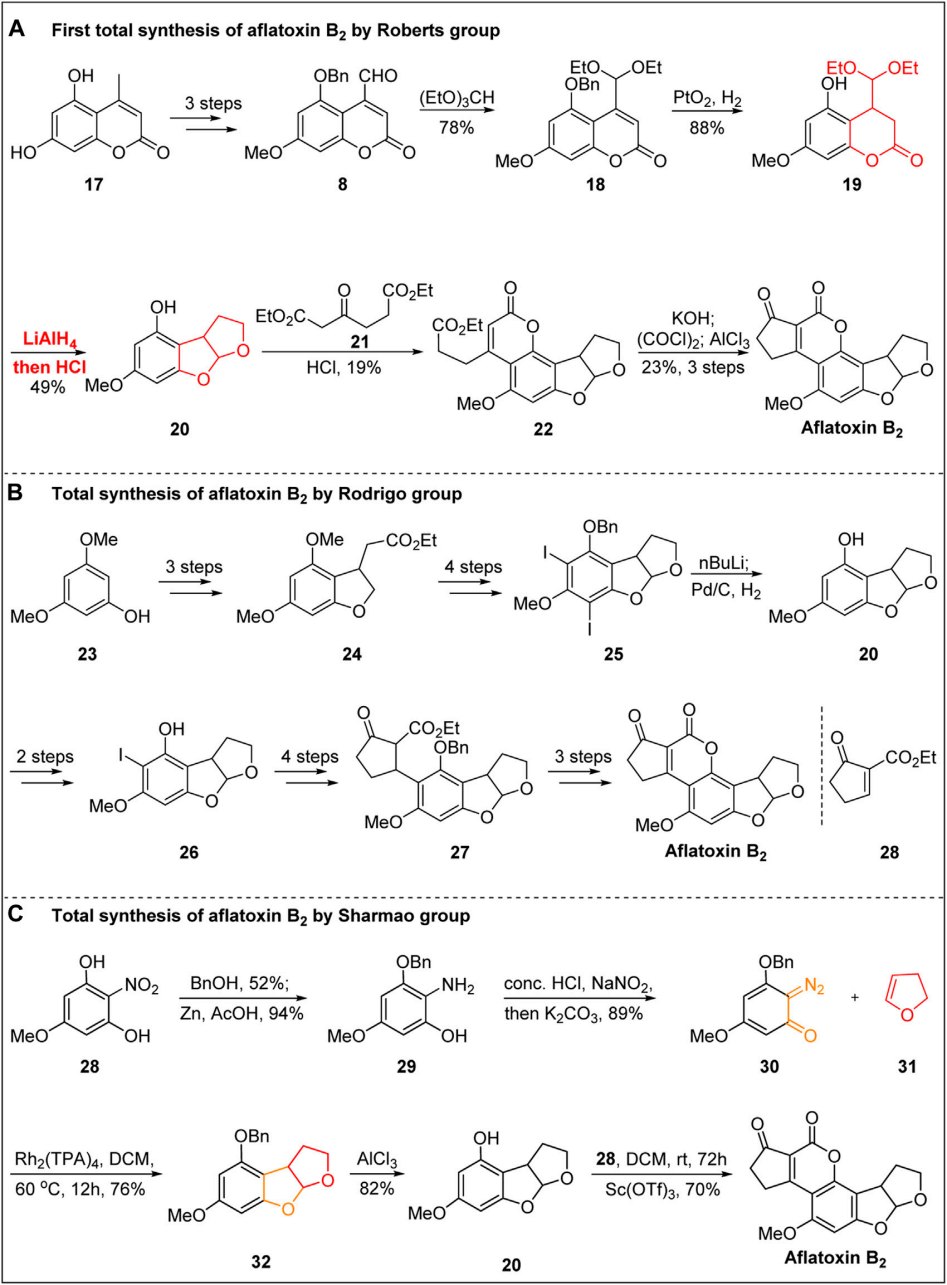
Total synthesis of (±)-aflatoxin B_2_.

Aflatoxin B_1_ also attracted the attention of Roberts’ group because of its unique chemical structure, although Büchi’s group was first to report the total synthesis of aflatoxin B_1_. Roberts’ group (Roberts et al., 1968) then switched their focus to the total synthesis of aflatoxin B_2_. In 1967, the total synthesis of aflatoxin B_2_ in 10 steps was reported for the first time, as shown in Figure 3A.

Using the same strategy reported by Büchi’s group, starting from the coumarin **17**, the coumarin intermediate **8** containing an aldehyde group was obtained by selective methylation, benzylation, and allyl oxidation under the action of SeO_2_. After the aldehyde group was protected, the benzyl protecting group was removed, and the double bond was hydrogenated under the action of Adam’s catalyst. Subsequently, under the action of LiAlH_4_, the ester group was reduced to an alcohol, and the aldehyde group was released in the presence of hydrochloric acid. The intramolecular acetal was spontaneously generated, and the synthesis of **20** was achieved. The first synthesis of aflatoxin **B2** was then achieved by Pechmann condensation and Friedel–Crafts acylation using the same strategy reported by Büchi’s group.

In 1988, Rodrigo’s group (Weeratunga et al., 1988) started to the total synthesis of (±)-aflatoxin B_2_ starting from 3,5-dimethoxyphenol **23** and realized the construction of the B-ring through three steps: iodine substitution, alkylation, and intramolecular addition. The C-ring skeleton was then realized by reduction, selective demethylation, iodination, benzyl protection, and cyclization. After the deiodination and debenzyl reaction, the intermediate **20** was synthesized in 4% total yield.

In 1990, Rodrigo’s group (Horne et al., 1990) reported the second total synthesis of aflatoxin B_2_ after two years of trying and failing (Figure 3B). From the advanced intermediate **20**, the key precursor **26** was obtained through diiodization, selective deiodization, benzyl protection of phenolic OH, lithium halide exchange, and transfer metallization. The second synthesis of aflatoxin B_2_ was then completed via the 1,4-addition of unsaturated cyclopentanone **28**, removal of the benzyl protective group, hydrolysis of the ester group under acidic conditions, spontaneous esterification, and DDQ oxidation. The total yield of aflatoxin B_2_ in the above nine linear steps was 2.5%.

In 2021, an efficient approach for the total synthesis of aflatoxin B_2_ was described by Sharmao’s group (Paymode and Sharma, 2021) (Figure 3C). The key step involved in this synthesis is the Rh-catalyzed [3 + 2]-annulation of ortho-diazoquinone with enol ether. The key diazoquinone precursor **30** was obtained after mono-benzylation, the reduction of the nitro group, and diazotization. The key Rh-catalyzed [3 + 2]-annulation of diazoquinone **30** with enol ether **31** was then carried out in the presence of Rh_2_(OAc)_4_ in DCM, resulting in the annulation product **32**. The advanced intermediate **20** was then obtained after deprotecting the benzyl group using AlCl_3_. Finally, in the presence of the Lewis acid Sc(OTf)_3_ in DCM, as reported by Zu’s group (Wang and Zu, 2019), the total synthesis of aflatoxin **B2** was completed via Pechmann-type annulation and aerobic oxidation.

### 2.3 First Total Synthesis of (±)-Aflatoxin M_1_ by Büchi Group

**FIGURE 4:**
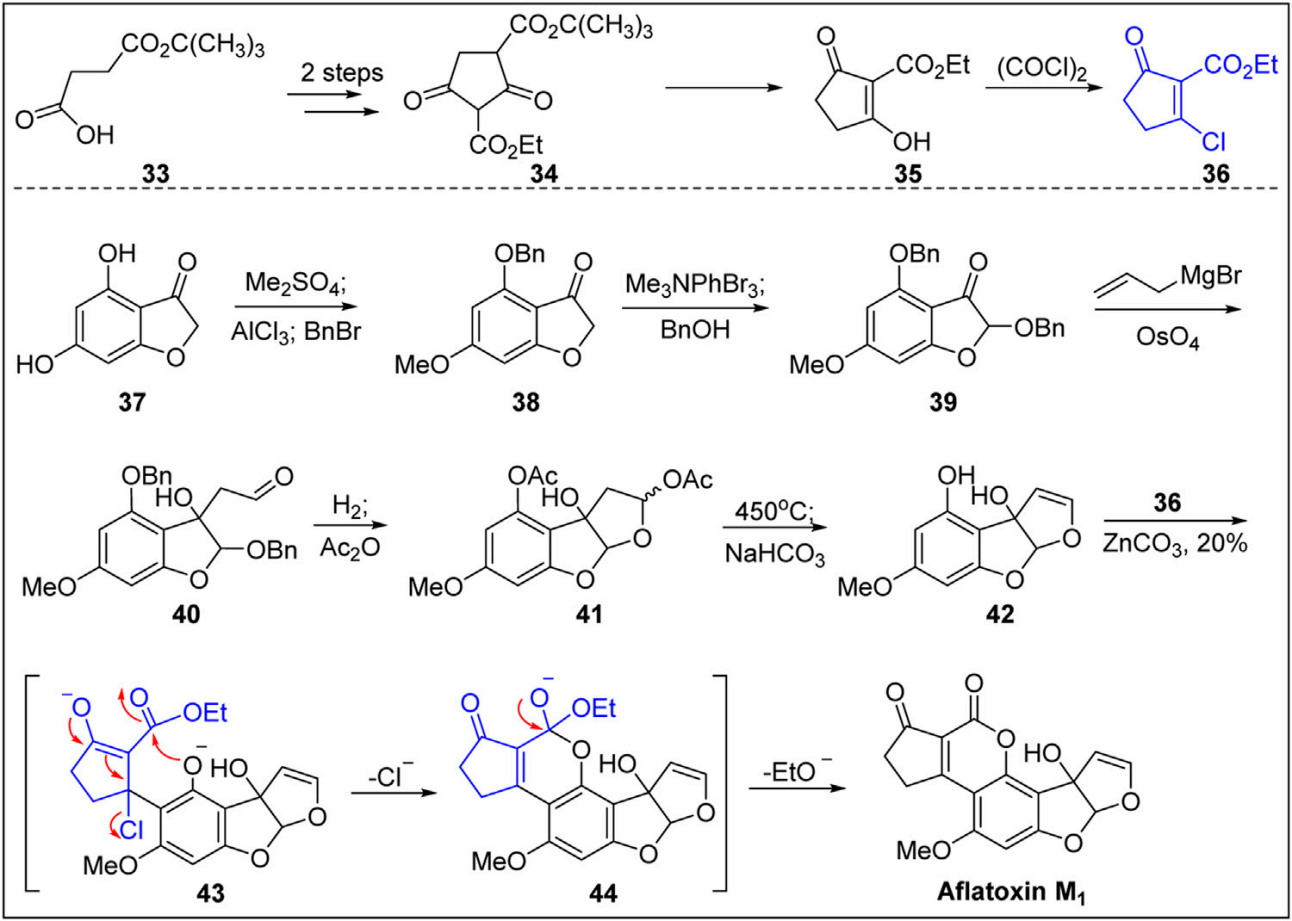
First total synthesis of (±)-aflatoxin M_1_ by Büchi group.

In 1969, Büchi’s group (Büchi and Weinreb, 1969) reported the first chemical total synthesis of aflatoxin M_1_, as shown in Figure 4. Based on the structural characteristic of aflatoxin M_1_, the dihydroxybenzofuranone **37** with a B-ring structure was used as the starting material, which created the conditions for the introduction of hydroxyl group and avoided the problem of constructing the B-ring. The hydroxyl-protected benzo furanone **38** was obtained after dimethylation, selective demethylation, and benzylation. The aldehyde **40** was then obtained via bromination (α-carbonyl group), substitution of benzyl alcohol, addition of allyl magnesium bromide to the ketone carbonyl group, and the oxidative fracturing of the double bond using osmium tetroxide. Two benzyl protection groups were removed by hydrogenation followed by the acylation of the phenol and hemiacetal hydroxyl groups and high-temperature pyrolysis (450°C), resulting in the construction of the C-ring double bond.

The free phenolic hydroxyl group in the A-ring was released via hydrolysis in the presence of weak base. The free phenolic compound **42** was then reacted with chlorinated unsaturated cyclopentanone **36** under the action of ZnCO_3_ to efficiently construct a D,E-bicyclic compound. The specific reaction process was as follows. First, under the catalysis of Zn ion, the 1,4-addition reaction took place smoothly between the phenolic OH group in the ortho-position and the unsaturated carbonyl substrate **36**. Next, the chloride anion as the leaving group left to form the unsaturated carbonyl intermediate. Finally, under the action of ZnCO_3_, the phenolic OH group underwent transesterification to produce ethyl ester, thereby completing the first total synthesis of aflatoxin M_1_. This procedure represents a new solution for the synthesis of aflatoxins.

### 2.4 First Total Synthesis of (±)-Aflatoxin G_1_ by Büchi Group

**FIGURE 5:**
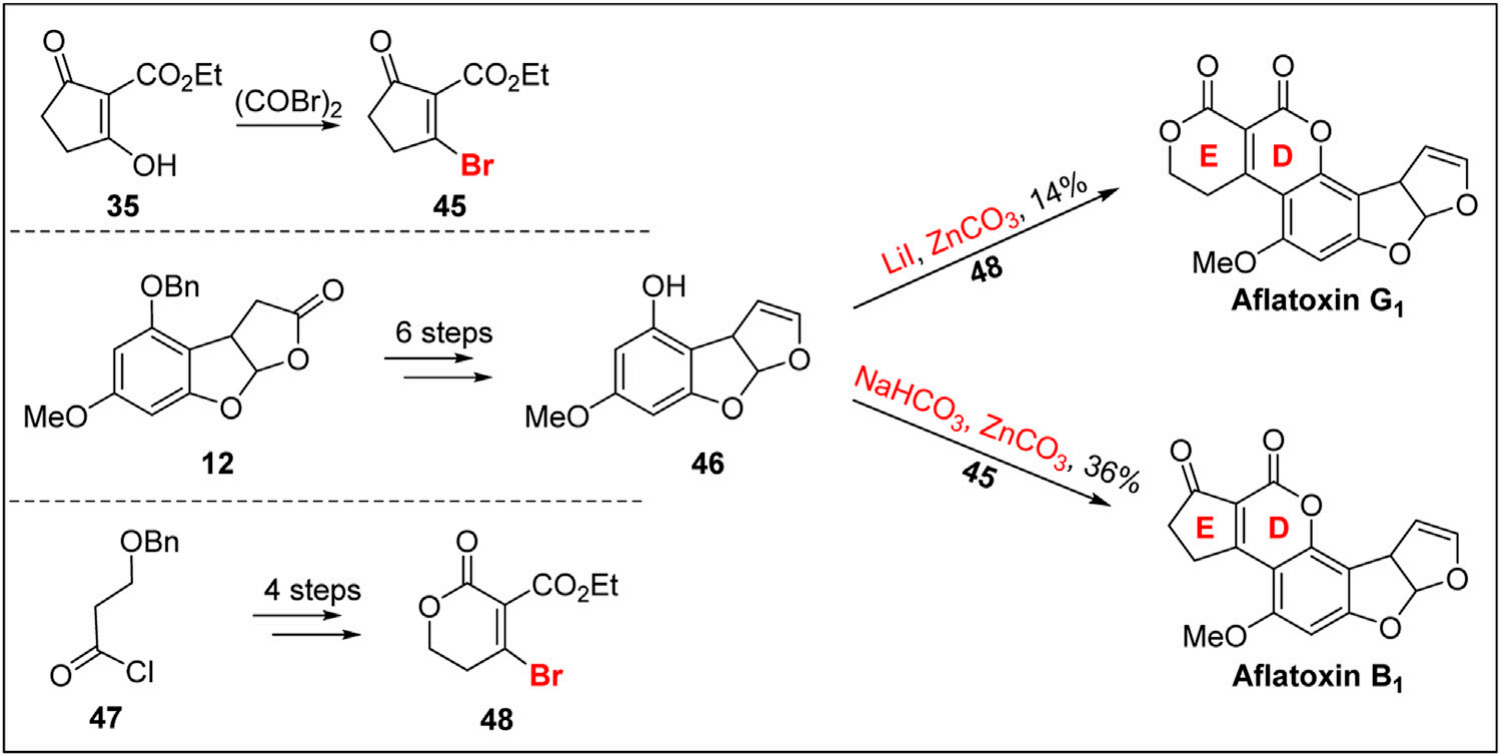
First total synthesis of (±)-aflatoxin G_1_ by Büchi group.

In 1971, Büchi’s group (Büchi and Weinreb, 1971) optimized the synthesis of aflatoxin B_1_ and completed the first total synthesis of aflatoxin G_1_ via a sequence of 1,4-addition, elimination, and transesterification reactions, as shown in Figure 5.

The synthesis of the advanced intermediate **46** was carried out from intermediate **12** through six steps: DIBAL-H reduction, acylation, hydrogenation of the benzyl group, acylation of the phenolic OH group, pyrolysis at high temperature (400°C), and deacylation. Finally, using the more active bromo-unsaturated cyclopentanone **45**, the D,E-bicyclic compound was formed in one step under reflux with ZnCO_3_ (130 equiv.). This method achieved the second-generation synthesis of aflatoxin B1 and provided an important reference for Corey’s group to synthesize aflatoxin B_2_.

In addition, Büchi’s group reported the bromo-unsaturated cyclopentanone **45** to the bromo-unsaturated caprolactone **48**. Based on this strategy, Büchi’s group achieved the chemical total synthesis of aflatoxin G_2_ for the first time, although the operation is complicated, and the yield was only 14%. This marks an important breakthrough in the synthesis of the G class of aflatoxins.

## 3 Asymmetric Total of Aflatoxins

### 3.1 First Asymmetric Total Synthesis of Aflatoxin B_1_ by Trost Group

**FIGURE 6:**
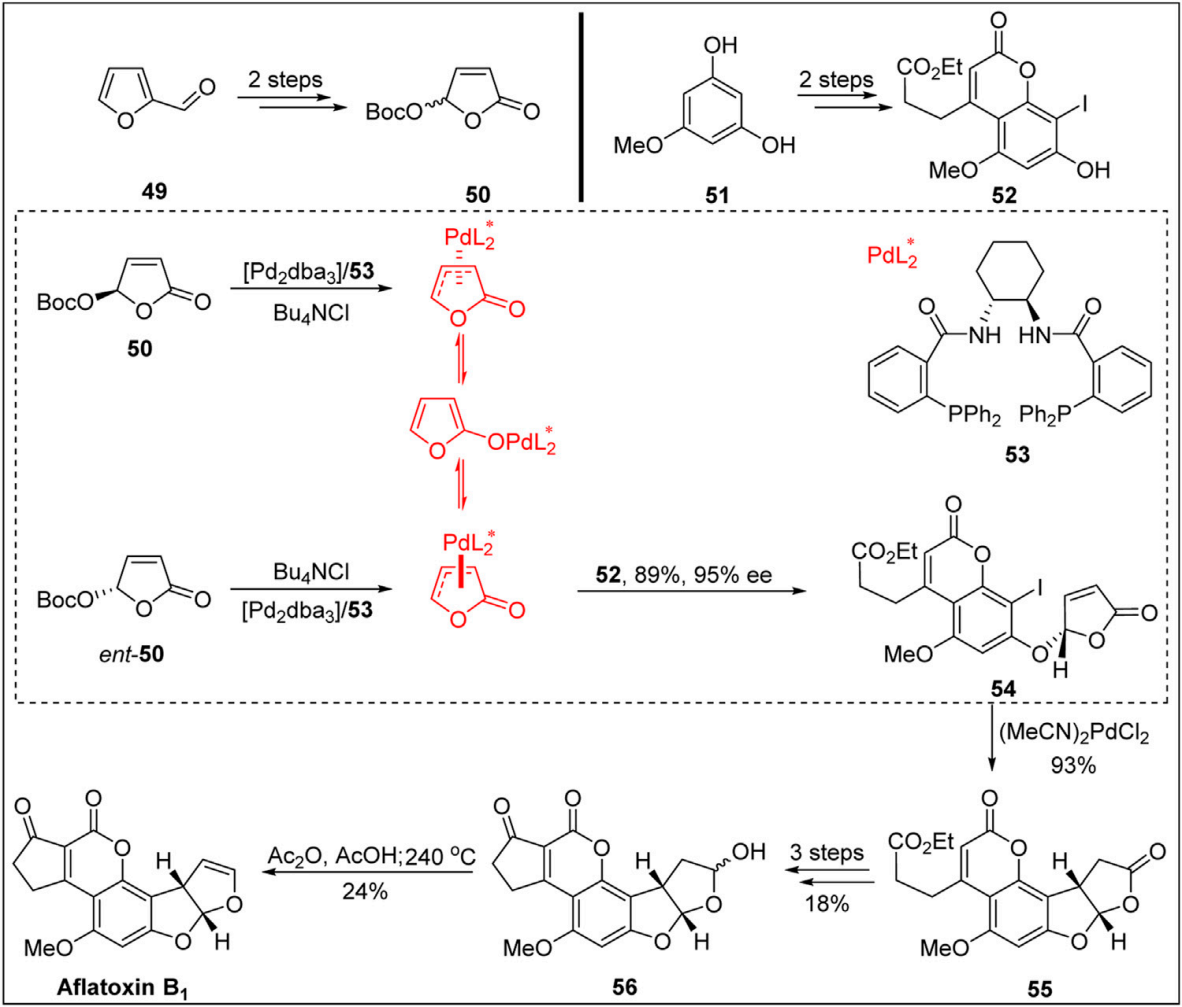
First asymmetric total synthesis of aflatoxin B_1_ by Trost group.

In 2003, Trost’s group (Trost and Toste, 2003) achieved the first chemical synthesis of aflatoxin B_1_ with high enantioselectivity via Pd-catalyzed dynamic kinetic asymmetric transformation (DYKAT), as shown in Figure 6.

Based on the work of the Buchi and Roberts groups, the coumarin precursor was constructed via Pechmann reaction from 5-methoxy-m-catechol **51** and β-keto ester. The key precursor **52** was then obtained via regionally selective iodination in the presence of ICl. Next, Trost’s group used their previously developed method (namely, the dynamic asymmetric transformation reaction of iodide and lactone under palladium catalysis) to construct the chiral center of the B-ring. The coupling product, which was obtained in high yield and with an excellent *ee* value, was subjected to intramolecular reduction via Heck reaction under standard conditions. The chiral center of the BC-ring was constructed with a high *ee* value, and the coumarin product **55** with an ABCD four-ring skeleton was obtained. In a later work, the Büchi group achieved the construction of the E-ring through a related transformation and then completed the chiral synthesis of aflatoxin B_2a_ **56**) via the selective reduction of the C-ring. Finally, aflatoxin B_1_ was synthesized via the acylation of the hemiacetal hydroxyl group followed by pyrolysis at 240 °C. The total yield of aflatoxin B_1_ in nine linear steps was 1.6%.

### 3.2 First Asymmetric Total Synthesis of Aflatoxin B_2_ by Corey Group

**FIGURE 7:**
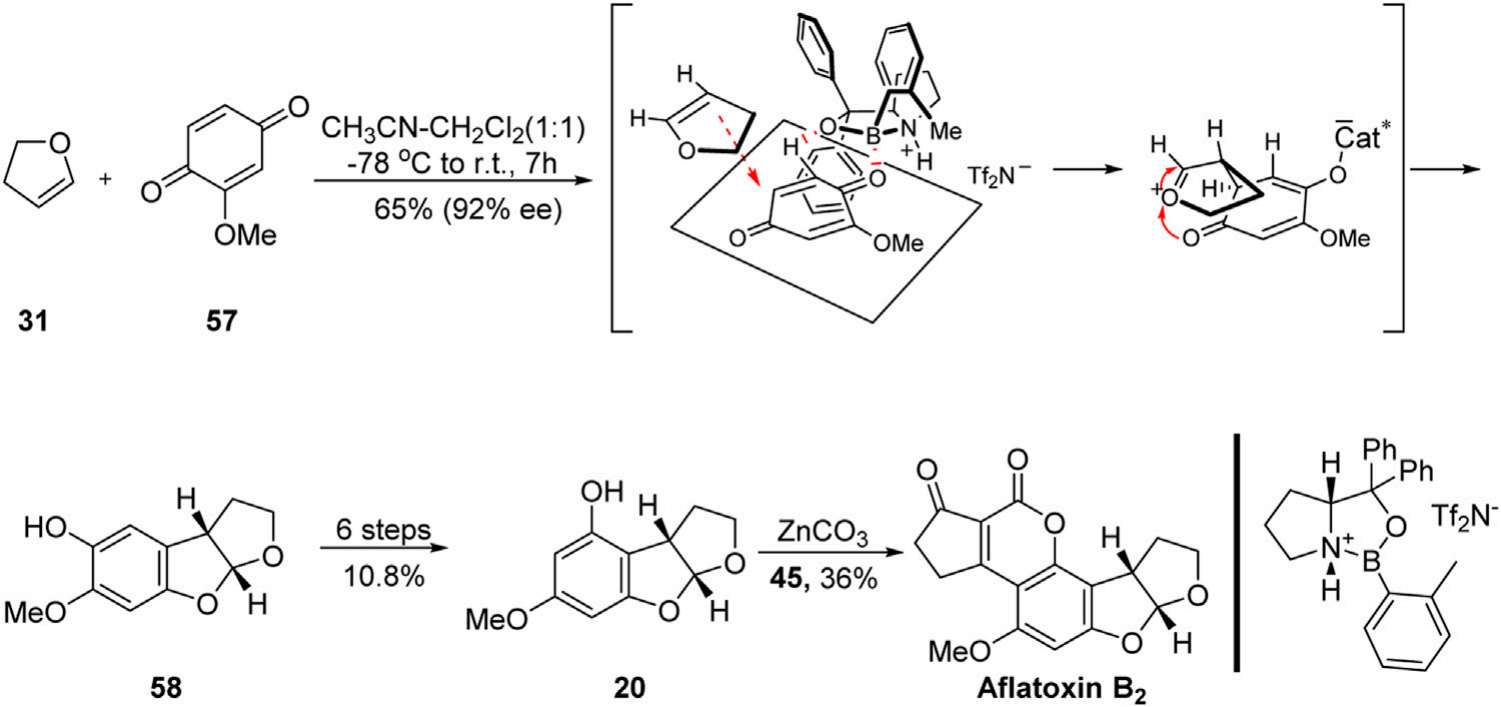
First asymmetric total synthesis of aflatoxin B_2_ by Corey group.

In 2005, inspired by the excellent work of the Büchi and Noland groups (Büchi and Weinreb, 1971; Noland and Kedrowski, 2000), Corey’s group (Zhou and Corey, 2005) reported the first asymmetric synthesis of aflatoxin B_2_ based on the use of several advanced intermediates, as shown in Figure 7. The key intermediate **58** was obtained with high efficiency from dihydrofuran **31** and 1,4-benzoquinone **57** based on a highly enantioselective [3 + 2] cycloaddition catalyzed by an organoboron reagent. The highly efficient formation of the ABC ring system was achieved in only one step.

It should be noted that the key precursor **58** obtained using this method was not a good match for the A-ring of aflatoxin B_2_. The authors then followed the synthetic strategy of Noland’s group (Noland and Kedrowski, 2000) (that is, Friedel–Crafts acylation, hydroxyl protection, 1,2-addition, DMP oxidation, oxygen insertion, saponification, and reduction) to obtain the advanced precursor **20**. Subsequently, they synthesized the DE-ring of aflatoxin B_2_ using the reaction conditions employed by Büchi’s group (Büchi and Weinreb, 1971). The first total synthesis of (+)-aflatoxin B_2_ was achieved in 2.5% yield through eight linear steps.

### 3.3 Asymmetric Total Synthesis of Aflatoxin B_2_ by Zu Group

**FIGURE 8:**
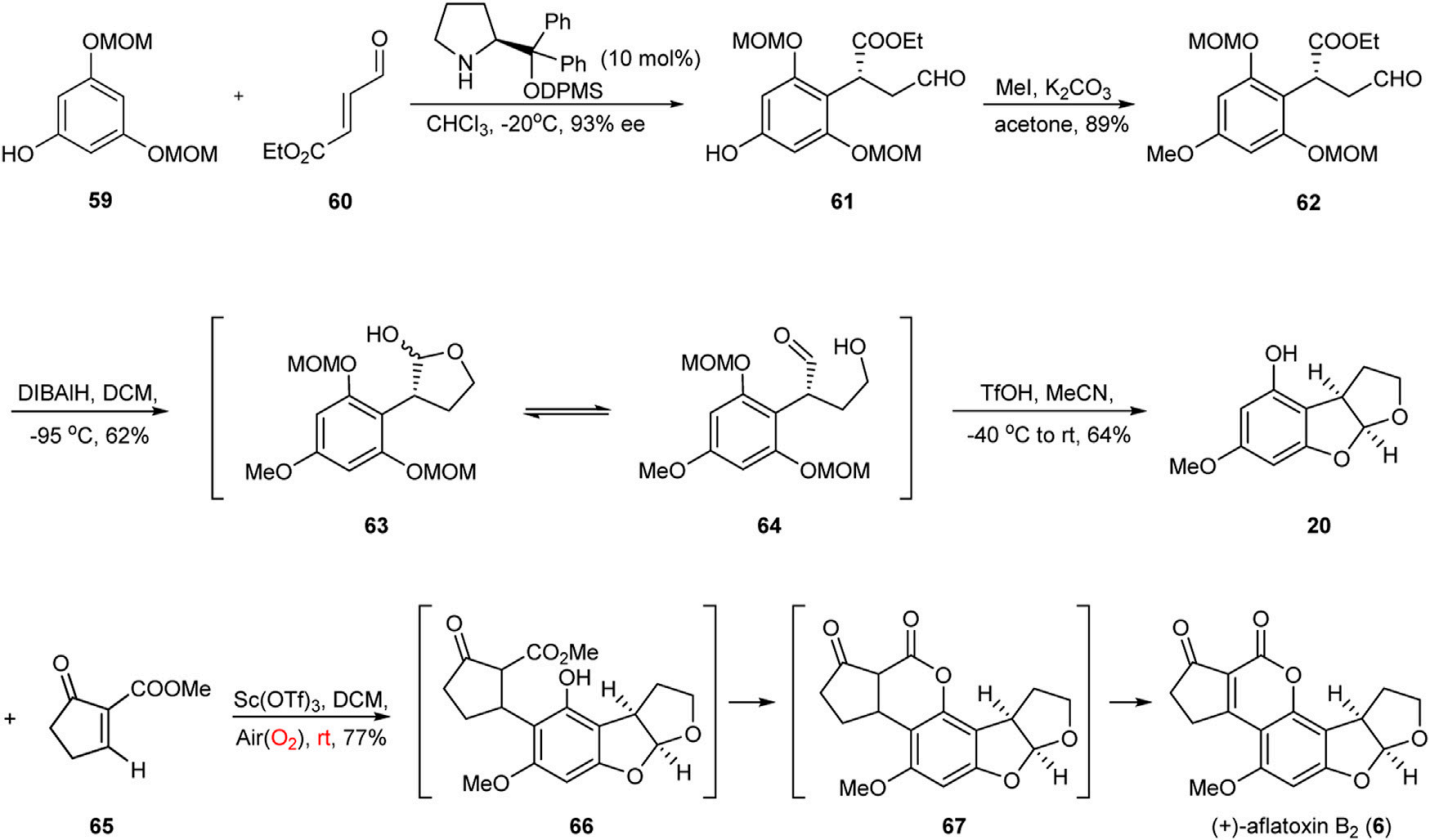
Asymmetric total synthesis of aflatoxin B_2_ by Zu group.

In recent years, green and facile single-pot reactions have received considerable attention in the field of chemistry because they can give rise to complex structures in few synthetic steps and with simple starting materials (Newhouse et al., 2009; Trost, 1991; István T. Horváth and Anastas, 2007; Wender et al., 2008; Hayashi, 2016). In 2019, an efficient Sc(OTf)_3_-promoted green and facile single-pot reaction involving 1,4-addition, intramolecular lactonization, and spontaneous aerobic oxidation was developed by Zu’s group (Wang and Zu, 2019) to synthesize the DE-ring system of aflatoxin B_2_ (Figure 8).

The synthesis started from the known phloroglucinol derivative **59** and the α,β-unsaturated aldehyde **60**. The enantioselective Friedel–Crafts alkylation of **59** with **60** provided the alkylation product **61**, which was methylated with MeI in acetone to provide intermediate **62**. It was gratifying to find that the partial reduction of the intermediate **62** with DIBALH in DCM at −95 °C generated hemiacetal **63** and hydroxyl aldehyde **64** as an inseparable mixture in 62% yield. In the presence of TfOH in MeCN, the tricycle **20** was obtained via the cleavage of the MOM groups followed by intramolecular cyclization. Having successfully assembled tricycle **20**, the group turned toward the final stage of the total synthesis of aflatoxin B_2_: the Sc(OTf)_3_-promoted one-pot sequential reaction. Finally, they successfully completed the required conversion in 77% yield using the Lewis acid Sc(OTf)_3_, thereby achieving the asymmetric chemical total synthesis of (+)-aflatoxin B_2_ with excellent atom-, redox-, and step-economy. This work also demonstrates that the application potential of the new developed strategy for the construction of benzyl chiral centers in the synthesis of complex molecules.

## 4 Formal Total Synthesis of (±)-Aflatoxins

### 4.1 Formal Total Synthesis of Aflatoxin B_1_ by Snieckus Group

**FIGURE 9:**
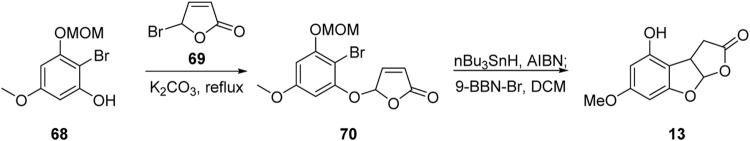
Formal total synthesis of aflatoxin B_1_ by Snieckus group.

In 1988, Snieckus’ group (Sloan et al., 1988) reported the formal total synthesis of aflatoxin B_1_ based on radical cyclization, as shown in Figure 9. The radical precursor 70 was obtained via substitution reaction from o-bromophenol **68** and bromobutenolactone **69**. The B-ring skeleton was then successfully constructed by intramolecular 1,4-addition mediated by free radicals. Finally, the MOM protecting group was removed to obtain the advanced intermediate **13**. According to Büchi’s strategy, the aflatoxin B_1_ was successfully synthesized.

### 4.2 Formal Total Synthesis of Aflatoxin B_2_ by Rapoport Group

**FIGURE 10:**
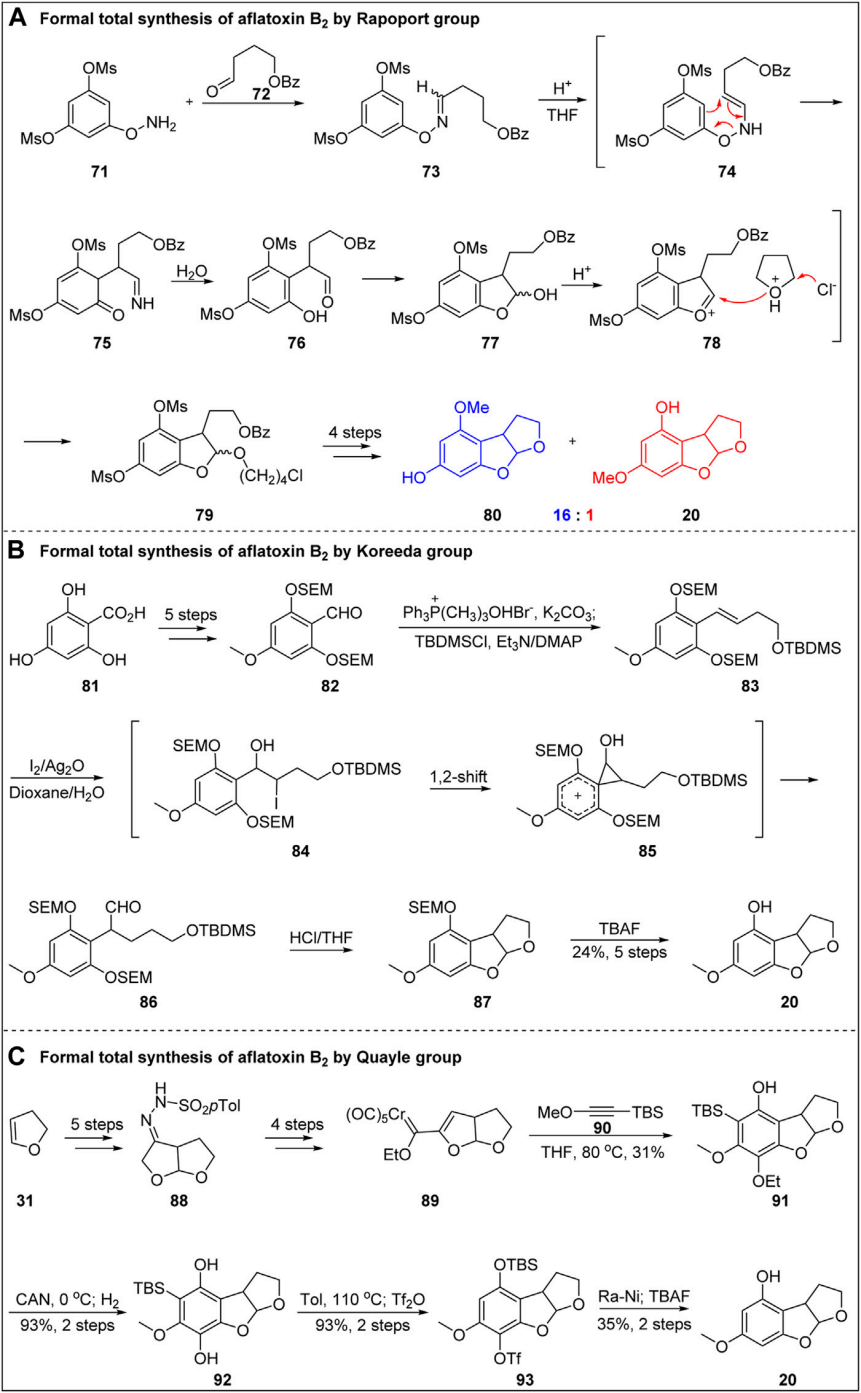
Formal total synthesis of aflatoxin B_2_.

In 1986, Rapoport’s group (Castellino and Rapoport, 1986; Civitello and Rapoport, 1994) formally synthesized aflatoxin B_2_ via Oxaza–Cope rearrangement, as shown in Figure 10A. Hydroxylamine **71** and aldehyde **72** were condensed to obtain oxime compounds, which were refluxed in a sealing tube containing 3.9 M HCl in tetrahydrofuran for 24 h followed by Oxaza–Cope rearrangement, imine hydrolysis, tetrahydrofuran cleavage by chlorine, and spontaneous addition. Subsequently, one methyl sulfonyl group was removed by lithium hydroxide hydrolysis, and the C-ring was constructed under the catalysis of *p*-toluenesulfonic acid. The isomers **80** and **20** were then obtained in a 16:1 ratio. The formal total synthesis of aflatoxin B_2_ was achieved in six steps with a total yield of 2.9%.

In 1993, Koreeda’s group (Koreeda et al., 1993) formally synthesized aflatoxin B_2_ through Kikuchi rearrangement, as shown in Figure 10B. Starting from benzoic acid **81**, aldehyde **82** was obtained by esterification of carboxylic acid and selective methylation of phenol, followed by protection of phenol OH with SEM, reduction of ester group and subsequent oxidation. Then, they introduced side chain compound **83** containing double bond by Wittig reaction. The key precursor **83** was subjected to Kikuchi rearrangement in iodine, silver oxide, and dioxane/water system, resulting in chain-branching 1,2-migration product **86**. After the removal of protective group and tandem cyclization reaction, the construction of advanced intermediate **20** was completed. It is worth to mention that Kikuchi rearrangement, as a newly developed method, plays an important role in the formal synthesis of aflatoxin B_2_, which confirms that the advance of the method has important promoting value to the total synthesis.

In 2006, Quayle’s group (Eastham et al., 2006; Eastham et al., 2008) reported an efficient method for the formal total synthesis of aflatoxin B_2_ via Wulff-Dötz reaction, as shown in Figure 10C. Starting from the C-ring compound dihydrofuran **31**, the B-ring skeleton was constructed by cobalt-mediated cyclization, and then **88** underwent a series of functional group transformations, such as ozone breaking and hydrazine to form a hydrazone, to obtain the key precursor of wulff-Dötz reaction. Then wulff-Dötz reaction of **89** with alkyneen **90** in THF was performed to obtain the construction of A-ring. Finally, the formal synthesis of aflatoxin B_2_ was successfully completed through simple transformation of **91**.

### 4.3 Formal Total Synthesis of Aflatoxin M_2_ by Kraus Group

**FIGURE 11:**
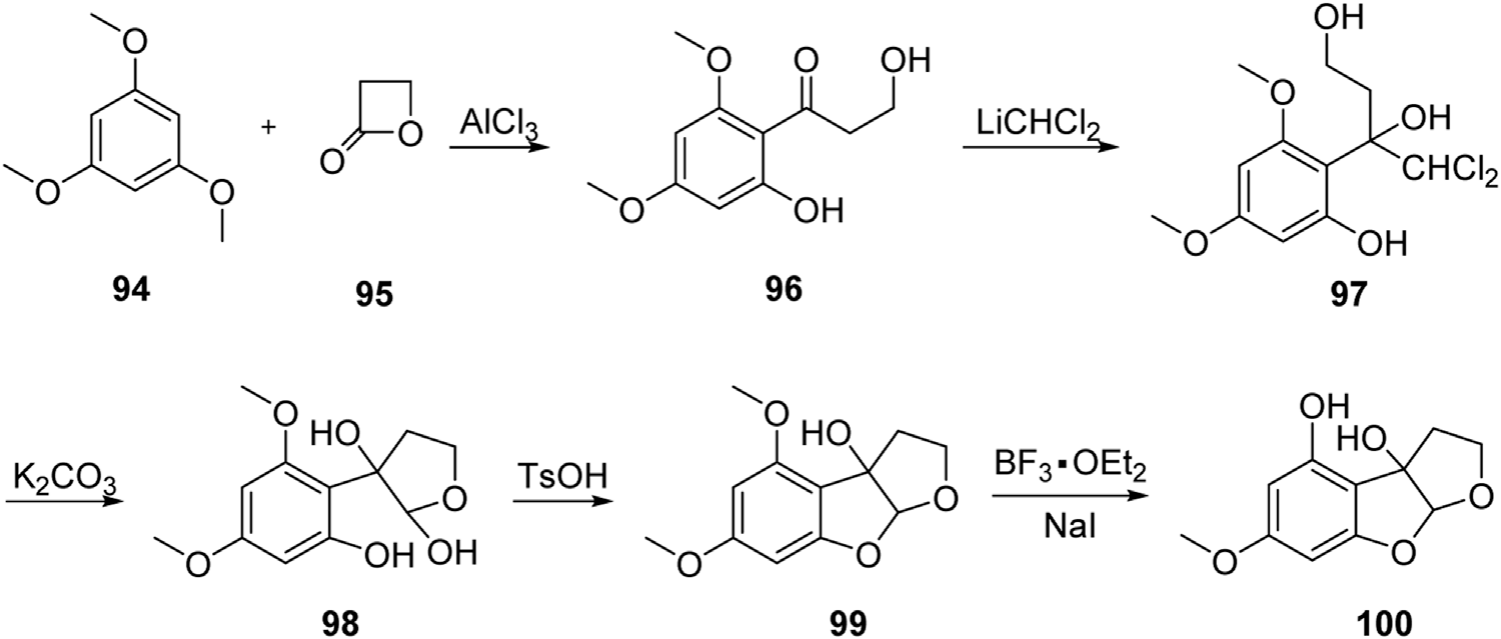
Formal total synthesis of aflatoxin M_2_ by Kraus group.

In 1999, Kraus’ group (Kraus and Wang, 1999) reported the first formal synthesis of aflatoxin M_2_ via the 1,2-addition of dichloromethyl lithium to carbonyl, as shown in Figure 11. The Friedel–Crafts acylation product **96** was obtained by the reaction of isotrimethoxylbenzene **94** with propanolactone **95** under the catalysis of AlCl_3_. Subsequently, **96** reacted with dichloromethyl lithium to obtain the key intermediate triol **97** via 1,2-addition. The hemiacetal intermediate **98** was hydrolyzed and cyclized under the action of potassium carbonate in isopropyl solution.

The B-ring was then constructed under the catalysis of TsOH. Finally, **99** was subjected to selective demethylation under the action of BF_3_•Et_2_O, and the advanced intermediate **100** was synthesized in five steps with a total yield of 26%. Later, the chemical synthesis of aflatoxin M_2_ was achieved based on Büchi’s method for the total synthesis of aflatoxin M_1_.

## 5 Asymmetric Formal Total Synthesis of Aflatoxins

### 5.1 Stereoselective Formal Total Synthesis of Aflatoxin B_1_ by Marino Group

**FIGURE 12:**
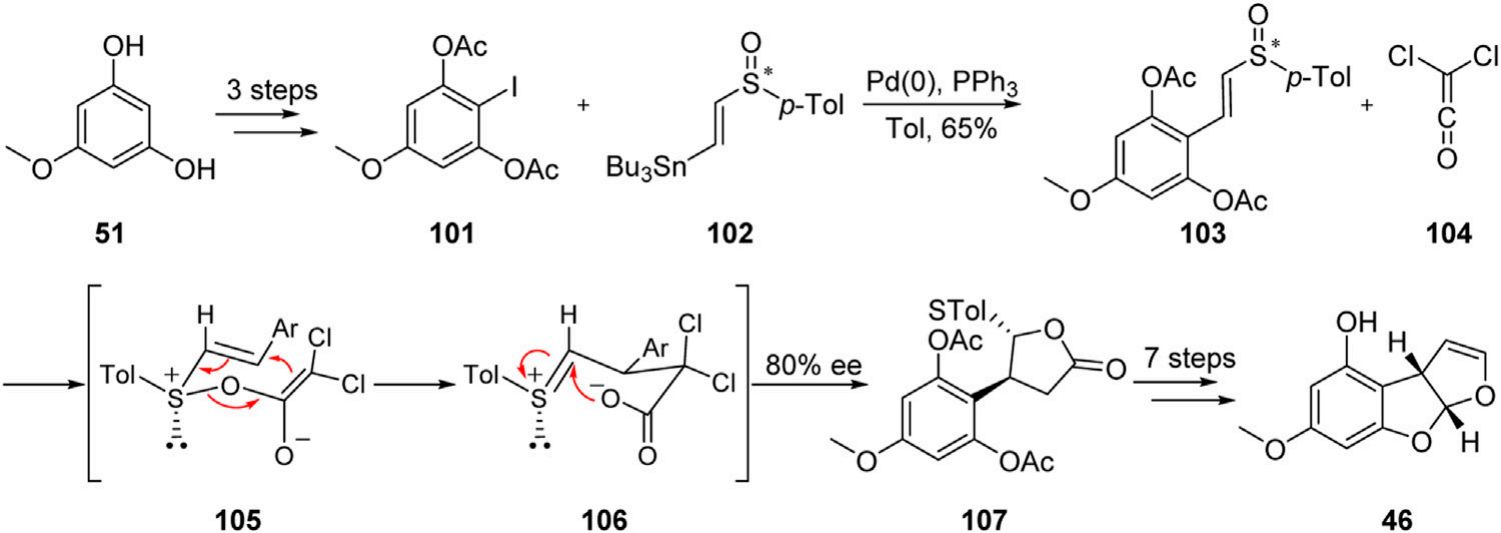
Stereoselective formal total synthesis of aflatoxin B_1_ by Marino group.

In 1993, the Marino group (Marino, 1993; Marino et al., 2011) described an efficient and stereoselective approach for the formal total synthesis of aflatoxin B_1_ with 80% *ee*. This approach is characterized by the [3,3]-σ rearrangement of chiral vinyl sulfoxide **103** and dichloroethylene ketone **104**, as shown in Figure 12.

The key sulfoxide precursor **101** was obtained from diphenol **51** by acylation, iodization, and Stille coupling. The C-ring was then successfully constructed by [3,3]-σ rearrangement. Finally, the formal synthesis of aflatoxin B_1_ was successfully achieved through eight steps including deacylation and cyclization.

### 5.2 Stereoselective Formal Total Synthesis of Aflatoxin B_2_ by Shishido Group

**FIGURE 13:**
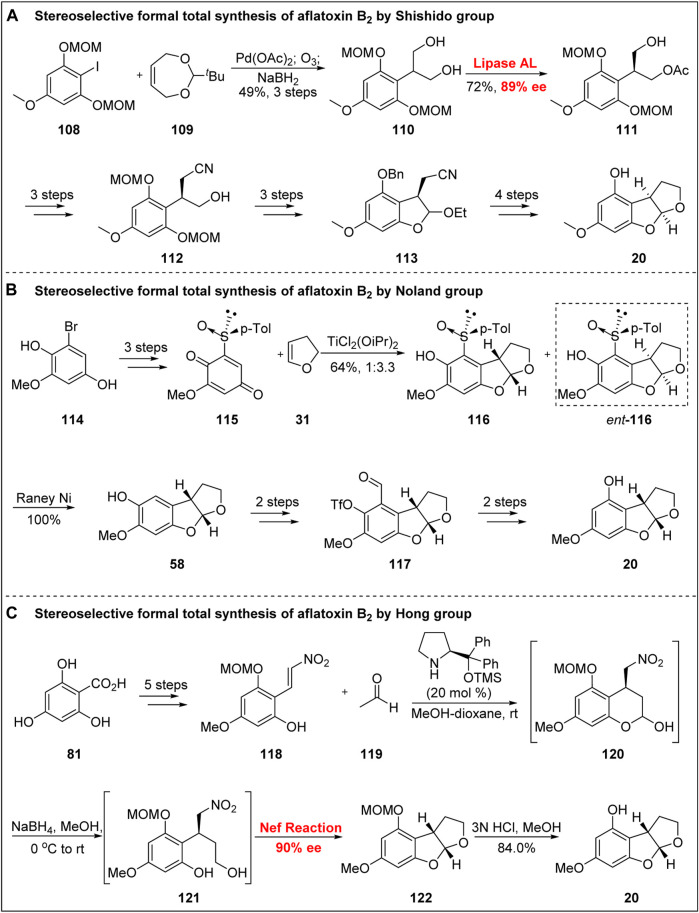
Stereoselective formal total synthesis of aflatoxin B_2_.

In 1997, Shishido’s group (Bando and Shishido, 1997) used the lipase-mediated asymmetric acetylation of prochiral diol compounds as the key strategy to accomplish the asymmetric formal synthesis of aflatoxin B_2_, as shown in Figure 13A. The coupling product was obtained from iodide **108** via Heck reaction. After ozonation and NaBH_4_ reduction, the diol **110** was obtained. After screening with a large number of lipases, the authors found that the lipase AL mediated the transfer of the ester group from *Achromobacter* sp., resulting in high yield with an *ee* of 89%. Compound **111** was then transformed into cyanide **112** by the introduction of a methyl sulfonyl group, cyanogen substitution, and deacylation. Subsequently, the B-ring was constructed via the oxidation of alcohol, the deprotection of phenol, and tandem cyclization. The benzylation of phenolic OH group, homeopathic reduction to alcohol after hydrolysis of cyanide. Next, under the action of TsOH, the C-ring was obtained through intramolecular cyclization. Finally, the asymmetric formal synthesis of aflatoxin B_2_ was realized via debenzylation.

In 2000, Noland’s group (Noland and Kedrowski, 2000) reported the asymmetric formal synthesis of aflatoxin B_2_ using chiral sulfoxide, as shown in Figure 13B. Based on the method developed by Andersen’s group (Andersen et al., 1964), a compound containing a sulfoxide subsidiary was synthesized from the known intermediate **114** after MOM protection. Under the action of CAN, the quinone sulfoxide intermediate **115** was obtained by oxidative demethylation. Under the catalysis of the Lewis acid TiCl_2_(OiPr)_2_, compounds **116** and ent-**116** were obtained in a ratio of 1:3.3, and the construction of the ABC tricyclic skeleton was realized. The sulfoxide auxiliary group was then removed by Laney nickel. After Duff reaction, the formylation product was obtained, and the phenol was esterified.

The functional group transformation of **117** was performed by Baeyer–Villiger oxidation, saponification, and reduction under the action of Laney nickel to obtain the precursor **20**. Notably, their synthetic strategy and particularly the late functional group transformation provided important ideas and inspiration for the asymmetric total synthesis of aflatoxin B_2_ by Corey’s group (Zhou and Corey, 2005).

In 2017, Hong’s group (Huang et al., 2017) reported the concise formal total synthesis of (-)-aflatoxin B_2_ based on the synthesis of an advanced intermediate of **20** in seven steps using an organic-catalyzed tandem one-pot reaction (Figure 13C). Starting from benzoic acid **81**, the key precursor **118** was obtained by using acetone to protect the carboxyl group and its o-phenol OH followed by selective methylation, MOM protection, reductive deprotection using DIBAL-H, and aldol reaction. Subsequently, using the organic catalytic tandem one-pot reaction developed by their own group, Hong’s group obtained the BC double-ring skeleton with excellent enantioselectivity. The specific sequence of the tandem reaction was as follows: first, under the action of Jørgensen catalyst and in the presence of acetic acid, the hemiacetal **120** was obtained with high *ee*; second, under the action of NaBH_4_, diol **121** was generated; finally, the BC double ring was constructed by Nef reaction. Subsequently, the asymmetric synthesis of **20** was completed by the removal of MOM.

## 6 Summary and Outlook

Given the broad implications that aflatoxins have for public health, considerable progress has been made in the total synthesis of aflatoxins since the 1960s. During the past 55 years, three enantioselective total syntheses have been described, including the first asymmetric total synthesis of aflatoxin B_1_ and B_2_ by the groups of Trost and Corey, and the second asymmetric total synthesis of aflatoxin B_2_ by the group of Zu. These works represent wonderful progress in the total synthesis of aflatoxins in terms of elegance, efficiency, and environmental friendliness.

**FIGURE 14:**
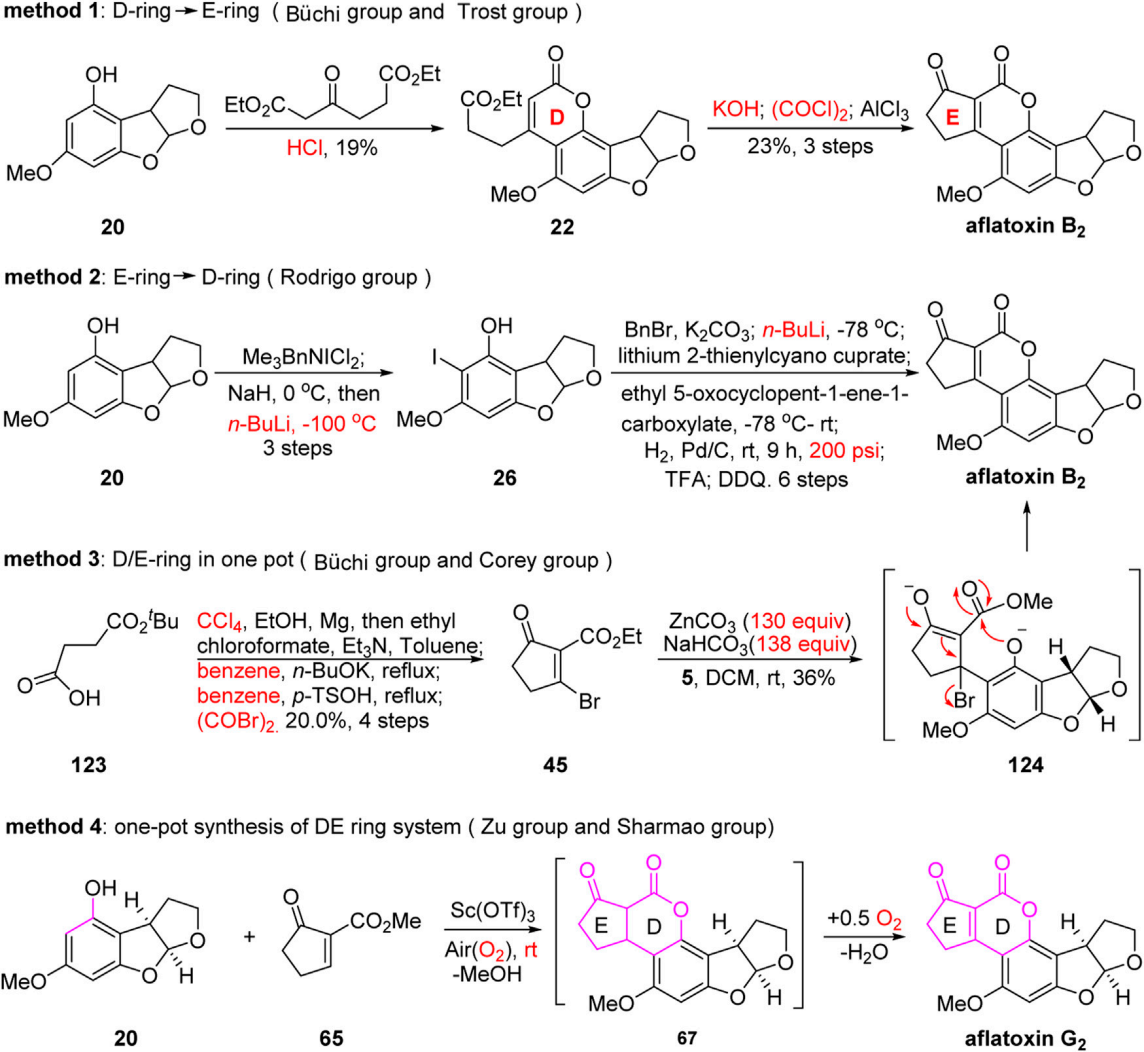
Four types of methods for the preparation of the DE ring system.

Most reported studies have focused on four types of methods. Taking the preparation of the DE ring system as an example (Figure 14), in 1966, the group of Büchi assembled the DE ring system via Pechmann condensation and Friedel–Crafts acylation in four consecutive steps with an extremely low overall yield (4.4% yield). Later, in 1971, Büchi’s group used a brominated five-membered cyclic ketone or six-membered lactone in the presence of ZnCO_3_ (130 equiv.) and NaHCO_3_ (138 equiv.) to successfully obtain the DE ring system in one step. However, it should be noted that the preparation of the brominated ketone or lactone requires four or five steps along with CCl_4_ and benzene as solvents, which is not ideal. In 1990, the group of Rodrigo assembled the DE ring system using a very different strategy from Büchi’s. Rodrigo’s approach involved transmetalation and addition with nine tedious consecutive steps, and both low temperature (−100°C) and high pressure (200 psi) were required. In 2019, the group of Zu developed a new Sc(OTf)_3_-promoted, green, facile, and economic single-pot strategy to synthesize the DE ring system of aflatoxins.

In brief, economic, facile, and green processes are being advanced as effective ways to not only form the key structural motifs of aflatoxins but also to circumvent tedious purification steps and promote environmental sustainability. Given the vital importance of this topic, the organic chemistry community should continue to invest efforts in developing new methods for the efficient total synthesis of aflatoxins.
